# The Coral Probiotics Village: An Underwater Laboratory to Tackle the Coral Reefs Crisis

**DOI:** 10.1002/ece3.71558

**Published:** 2025-07-04

**Authors:** Neus Garcias‐Bonet, Helena Villela, Francisca C. García, Gustavo A. S. Duarte, Nathalia Delgadillo‐Ordoñez, Inês Raimundo, Yusuf C. El‐Khaled, Erika P. Santoro, Morgan Bennett‐Smith, Brian O. Nieuwenhuis, João Curdia, Brian Zgliczynski, Clinton Edwards, Stuart Sandin, Eslam O. Osman, Ronell Sicat, Alexander Przybysz, Alexandre S. Rosado, Burton H. Jones, Francesca Benzoni, Michael L. Berumen, Khaled Salama, Shinkyu Park, Manuel Aranda, Carlos M. Duarte, Sebastian Schmidt‐Roach, Charlotte A. E. Hauser, Tadd Truscott, David J. Suggett, Christian R. Voolstra, Susana Carvalho, Raquel S. Peixoto

**Affiliations:** ^1^ Biological and Environmental Science and Engineering (BESE) King Abdullah University of Science and Technology (KAUST) Thuwal Saudi Arabia; ^2^ Department of Biology Boston University Boston Massachusetts USA; ^3^ University of California San Diego San Diego California USA; ^4^ Visualization Core Lab King Abdullah University of Science and Technology Thuwal Saudi Arabia; ^5^ Computer, Electrical and Mathematical Sciences and Engineering (CEMSE) King Abdullah University of Science and Technology (KAUST) Thuwal Saudi Arabia; ^6^ Laboratory for Nanomedicine, Computational Bioscience Research Center (CBRC) King Abdullah University of Science and Technology (KAUST) Thuwal Saudi Arabia; ^7^ Institute for Health Care Engineering Graz University of Technology (TU Graz) Graz Austria; ^8^ Department of Mechanical Engineering, Physical Science and Engineering (PSE) King Abdullah University of Science and Technology (KAUST) Thuwal Saudi Arabia; ^9^ KAUST Reefscape Restoration Initiative (KRRI) King Abdullah University of Science and Technology Thuwal Saudi Arabia; ^10^ Climate Change Cluster, Faculty of Science University of Technology Sydney Ultimo New South Wales Australia; ^11^ Department of Biology University of Konstanz Konstanz Germany

**Keywords:** beneficial microorganisms for corals, coral microbiome stewardship, coral reefs restoration, microbial‐based therapies, sustained environmental data observations, technology development

## Abstract

Restoration efforts are urgently needed for the conservation of coral reefs. Among emerging tools, the use of probiotics has shown promise in laboratory settings for improving coral resilience, but their validation under real‐world reef conditions remains limited. Here, we present the Coral Probiotics Village (CPV) as a novel and fully operational underwater research laboratory and a testing ground designed to support the in situ testing of microbial‐based coral restoration interventions. This manuscript describes the design, establishment, and scientific validation of the CPV, including continuous environmental monitoring, a summary of previously developed pilot trials of probiotic applications, and an integration of sensor networks, AI‐assisted reef monitoring, and autonomous technologies. We propose the CPV as a scalable model for integrated coral restoration science and suggest its replication as a tool to accelerate applied reef conservation efforts globally.

## Graphical abstract

The Coral Probiotics Village is the first underwater laboratory using an integrated and holistic approach to test innovative solutions to rehabilitate coral reefs, with a main focus on the use of microbial‐based therapies. The pillars of this initiative include the establishment of a reproducible platform for sustained environmental data observations, pioneering research, including in situ coral microbiome stewardship, technology development, restoration efforts, disease outbreaks, and invasive species surveillance, outreach, and education.

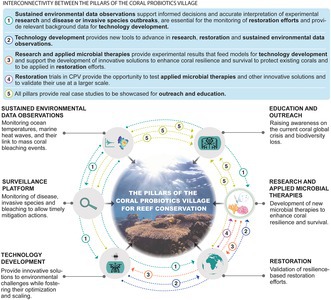

## 1 Introduction

Coral reefs are highly valuable ecosystems providing diverse ecosystem services (Costanza et al. 2014; Spalding et al. 2017) that support—directly or indirectly—almost a billion people worldwide (Sing Wong et al. 2022). However, coral reefs are globally declining at alarming rates due to anthropogenic climate change (Hughes et al. 2003, 2018) and local impacts (Donovan et al. 2021). Global warming, in particular, is increasing the intensity and frequency of bleaching events, reducing recovery time between severe bleaching events (van Hooidonk et al. 2016; Hughes et al. 2018; Reimer et al. 2024) and increasing the frequency of coral disease outbreaks (Maynard et al. 2015). Mitigating the impact of global warming by reducing atmospheric greenhouse gases and removing local stressors together with active restoration efforts are crucial for the conservation of coral reefs (e.g., Kleypas et al. 2021, Peixoto, Voolstra, Baums, et al. 2024).

Strategies to increase the resistance of existing and restored corals to forthcoming thermal stress are urgently needed to halt the current loss of coral reefs (Baums et al. 2019; Suggett and van Oppen 2022; Suggett et al. 2023; Peixoto, Voolstra, Stein, et al. 2024) and have been defined as “assisted coral reef restoration,” which is the integration of several technologies that can ensure existing and restored reefs are retained (Peixoto et al. 2025). Such strategies require close monitoring and well‐controlled pilot systems that allow for the risk assessment and validation of these approaches. Strategies to enhance resilience within restored coral populations include the active manipulation of corals to increase tolerance to heat and other stressors, and enhance their recovery rates by tackling different compartments of the coral holobiont: coral host, *Symbiodiniaceae* symbionts, and the microbiome (bacteria and viruses) (Voolstra et al. 2021; Bay et al. 2023).

The use of “Beneficial Microorganisms for Corals” (BMCs), or probiotics for corals, has been highlighted as an innovative strategy to improve coral health by promoting coral nutrition and growth/development or mitigating the negative effects of pathogens and toxic compounds (Peixoto et al. 2017, 2019, 2021; Voolstra et al. 2021, 2024; Thatcher et al. 2022). BMCs are native members of the healthy coral microbiome that contribute to a broad range of functions, including nutrient cycling, pathogen control, UV protection, mitigation of toxic compounds or stress, growth and early life development and can be applied as probiotics to increase the holobiont health and resilience (Peixoto et al. 2017, 2021). A well‐defined framework for the screening and selection of BMCs includes the isolation and purification of microorganisms from healthy coral samples, screening for beneficial traits by physiological tests and/or detection of molecular markers, exclusion of isolates exhibiting pathogenic traits, the taxonomic analysis of potential candidates and, finally, the assemblage of selected putative beneficial isolates (Peixoto et al. 2017). Genomic analysis can also expand and contribute to this selection (Doering et al. 2023; Rosado et al. 2023; Raimundo et al. 2024).

In the coral aquaculture industry, the application of probiotics can improve coral mass production by limiting diseases and improving nutrition to meet the high supply requirements needed for large‐scale coral reef restoration activities (Thatcher et al. 2022). Recent publications have demonstrated that the application of probiotics elicits coral microbiome restructuring processes and host metabolic changes that result in improved holobiont physiological parameters (Santoro et al. 2021), increased calcification and coral growth rates (Zhang et al. 2021; Moradi et al. 2023), mitigated bleaching and decreased coral mortality due to thermal stress (Rosado et al. 2019; Santoro et al. 2021; Li et al. 2023; Cardoso et al. 2024; de Breuyn et al. 2025), slowed disease progression (Ushijima et al. 2023), and mitigated stress responses to heat and pathogens (Rosado et al. 2019) or oil spills (dos Santos et al. 2015; Silva et al. 2021). Such evidence is based on experiments carried out on coral fragments in tanks under controlled laboratory conditions. Although this is a relatively new field of research, the growing evidence of the positive effects of probiotic treatments for corals in tank trials discussed above indicates that in situ validation is urgently needed (Garcias‐Bonet et al. 2023).

**FIGURE 1:**
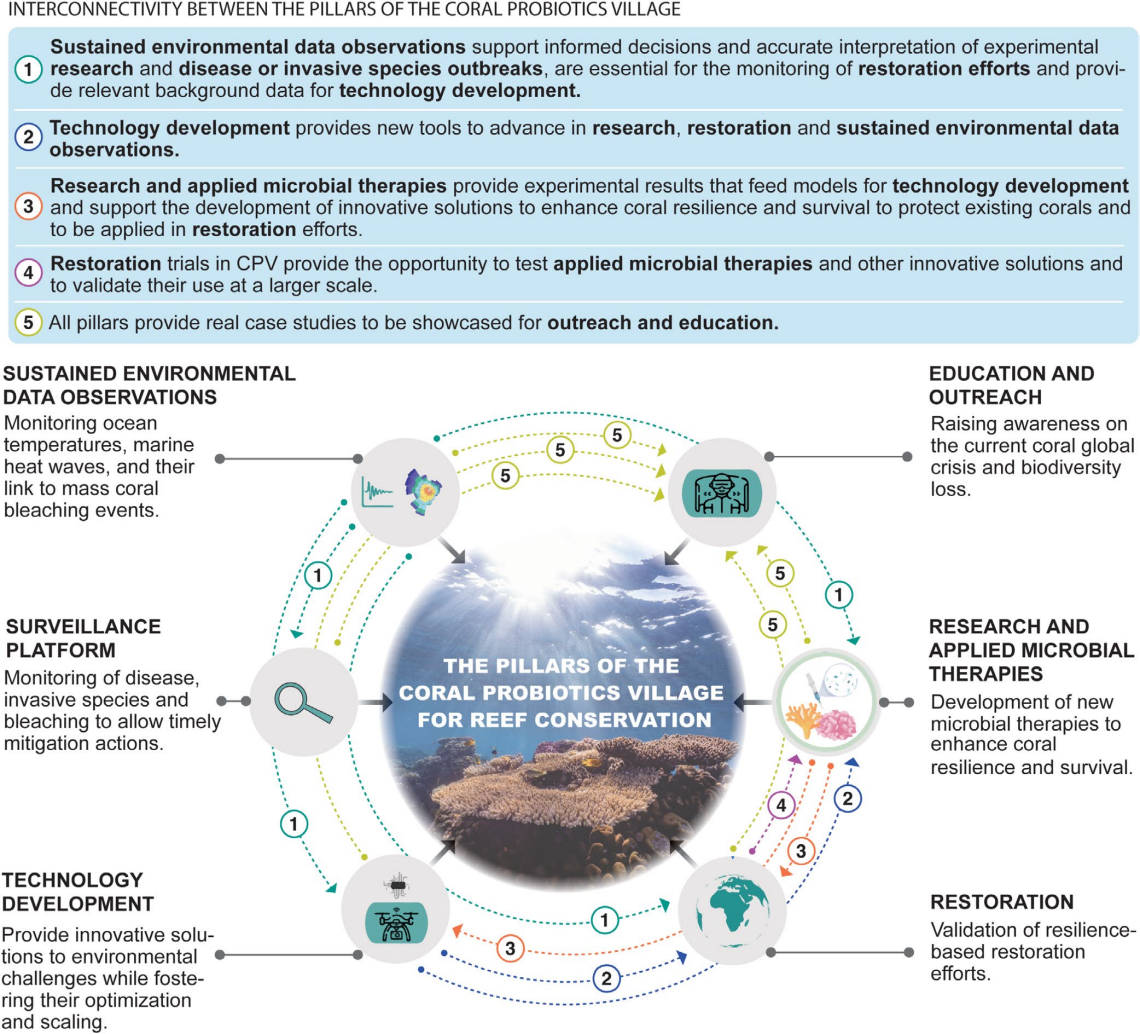
Main pillars of the Coral Probiotics Village highlighting the interconnectivity among them.

To address the urgent need for the development and implementation of scalable, in situ coral restoration using cutting‐edge solutions to enhance coral resilience (Peixoto, Voolstra, Stein, et al. 2024; Peixoto, Voolstra, Baums, et al. 2024), we established the Coral Probiotics Village (CPV). The CPV is the first permanent underwater laboratory designed to support experimental validation of microbial‐based reef restoration tools and other innovations under real‐world environmental conditions. This article presents the CPV as a research outcome in itself, describing its architecture, infrastructure, and scientific implementation. We detail the site's environmental monitoring platform, pilot studies on coral microbiome stewardship, and active testing of associated restoration technologies (Figure 1). Although some results presented here are reported for the first time, others have been previously published and are cited as components of the overall validation process. The summary of examples demonstrates the CPV's scientific functionality and offers a reproducible model for advancing microbial reef restoration. Our objective is to describe the structure, operational logic, and initial research outputs of the CPV to guide replication and global scaling of this approach.

## 2 Materials and Methods

### 2.1 The CPV

**FIGURE 2:**
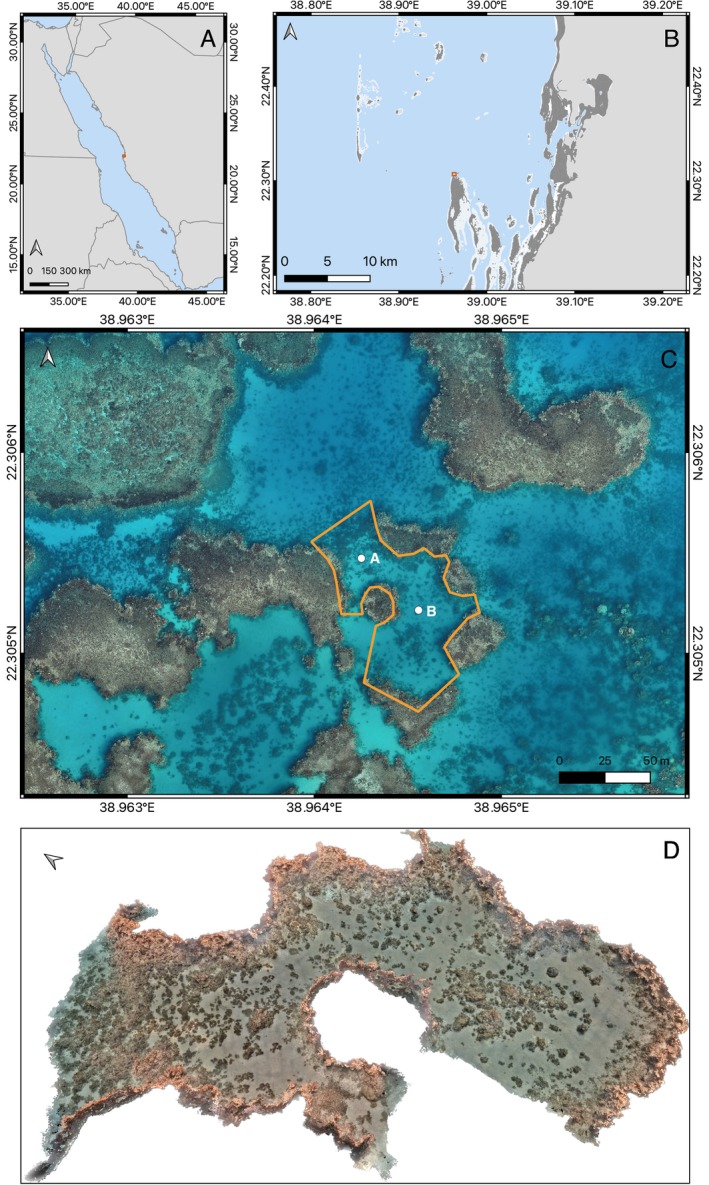
Maps showing the location of the study site (A, B) and aerial drone image taken on October 24, 2023 (C) of the Coral Probiotics Village indicating the area surveyed for the 3D model (orange polygon) with the two in situ data collection stations (A and B). Dark gray areas in the inset map correspond to coral reef distribution extracted from the Allen Coral Atlas (Allen Coral Atlas 2020). (D) The 3D photogrammetry model of the Coral Probiotics Village.

The CPV was established in June 2021 as a collaborative and multi‐disciplinary initiative to streamline efforts and resources on coral reef restoration and rehabilitation research, from several research groups at KAUST and colleagues from other universities. It is located in a sheltered shallow area at the northern end of Al Fahal Reef (22°18′18.4″N; 38°57′52.5″E), a midshore reef located at the edge of the continental shelf and about 15 km off the Saudi Arabian coasts, in the Red Sea (Figure 2A–C). The main area spans ~5000 m^2^ (0.5 ha) and has a maximum depth of 10 m. It comprises mixed reef habitats including walls, slopes, and coral heads in sand. The most dominant coral species are *Pocillopora* sp., *Acropora* spp., *Porites* spp., and *Millepora dichotoma* .

### 2.2 Environmental Monitoring and Site Characterization

We implemented in situ data recording stations in June 2021, acquiring high temporal resolution physico‐chemical variables (seawater temperature, salinity and currents) to fully characterize our study site, elucidate the seawater dynamics, and interpret the results from in situ coral microbiome stewardship experiments. Sustained environmental data observations support informed decisions and accurate interpretation of in situ experimental research. They are essential for the monitoring of restoration efforts and disease or invasive species outbreaks and provide relevant background data for technology development (Figure 1). Moreover, they provide a reference dataset for other reef studies in the region and expand the global sustained marine data observation platforms. In this sense, in situ daily and seasonal variations in seawater temperature are essential to mimic environmental reef conditions in experimental tanks, coral nursery tanks, and breeding conditioning systems for ex situ coral spawning.

In situ environmental seawater parameters (seawater temperature and salinity) and seawater currents have been continuously monitored since June 2021 using a Multiparameter CTD probe (Ocean Seven 310, Idronaut) and an Acoustic Doppler Current Profiler (ADCP, Aquadopp Profiler 1 MHz, Nortek) deployed in two locations (A and B, see Figure 2) within the main area. Instruments were deployed at 6.5 and 8 m deep in A and B locations, respectively, by attaching them to permanent frames (0.5 m × 0.5 m × 0.5 m) secured at the bottom in each site. Multiparameter CTD data collection was set to 10 measurements every 30 min. Multiparameter CTD probes were calibrated before each deployment following the ISO standards (A2LA certification to ISO/IEC 17025 standards) by the Coastal and Marine Resources Core Lab (KAUST). In order to avoid gaps in data collection, the instruments deployment dates overlapped and instruments were not retrieved simultaneously. No differences in temperature were observed between locations and therefore, temperature data are shown combining both stations. ADCP (seawater speed and direction) sampling frequency was set to 30‐min intervals on 0.5 m cell sizes, with a black distance of 0.2 m, resulting in a total of 13 cells (from 6.5 m depth to below the surface) in location A and a total of 16 cells (from 8 m depth to below the surface) in location B.

### 2.3 Digital Characterization of the CPV

To facilitate the characterization of the study area, digital representations of the CPV were generated at multiple scales using the large‐area imaging (LAI) approach (Edwards et al. 2023). LAI involves the collection of highly overlapping images that are processed to generate 3‐dimensional (3D) models and associated image products (e.g., ortho‐projected maps, digital elevation models, etc.). These products can be used for a variety of subsequent ecological analyses, as well as for visualization and digital exploration purposes.

In June 2022, the study area was divided into three sampling regions, each of which was surveyed over the course of a day, on three consecutive days. Surveys consisted of a team of SCUBA divers distributing a series of 50 cm calibration bars and reference tiles throughout the survey area to provide scaling and reference ground control points. The identification, depth, and location of each calibration bar and reference tile were recorded for later use during model construction and validation. Imagery was collected using Nikon d780 full‐frame cameras, outfitted with 24‐mm fixed focal length lenses mounted in underwater housings and mounted to an aluminum frame, with 1‐m spacing between cameras. Built‐in interval timers were used to capture images at a rate of 1 image per sec, and cameras were operated at constant speed to ensure adequate front‐to‐back overlap (60%–80%) among sequential images. Each region of the survey area was imaged with divers swimming the camera systems in a gridded pattern 1.5–2 m above the substrate following a double boustrophedonic pattern, with 1‐m spacing between passes to ensure necessary (60%–80%) side‐to‐side image overlap between adjacent passes. Commercially available photogrammetric software, Metashape Pro (Agisoft LLC., St. Petersburg, Russia) was used to create 3D models, generated as dense point clouds. 3D models were then exported to the custom software package Viscore (Petrovic et al. 2014) for further analysis and visualization purposes.

On October 24, 2023, an aerial map covering 20 ha around the CPV was created. To that end, the study area was surveyed with a DJI Phantom 4 Pro RTK at 100 m altitude. The drone followed a single boustrophedonic pattern with 90% frontal overlap and 75% lateral overlap. Shutter speed was set to 1/500 s, with the drone moving at 3.5 m/s. The resulting images were first geolocated with centimeter accuracy through post‐processing kinematics before they were processed with the Structure‐from‐Motion algorithms in Pix4Dmapper v4.8.4 to create an ortho‐projected map. An exhaustive description of the drone processing workflow adopted in this study can be found in Nieuwenhuis et al. (2022).

### 2.4 Data Analysis

For the CTD data, 10 measurements every 30 min were averaged first for each location, then daily mean, maximum and minimum values were calculated combining both locations. Relative Degree Heating Weeks and the warning alert levels were calculated for 2021, 2022 and 2023 following NOAA methodology detailed here: https://coralreefwatch.noaa.gov/product/5km/methodology.php. However, unlike the standard NOAA approach which utilizes satellite‐derived sea surface temperature, our analysis employed in situ seawater daily mean and maximum temperatures recorded between 6.5 and 8 m deep. Consequently, we propose the term “relative Degree Heating Weeks” (rDHW) to denote these locality‐specific measurements, which reflect bottom temperature conditions rather than surface readings. It is important to note that while rDHW is derived using NOAA's framework, the data used and, therefore, the results, are not directly comparable with those reported in NOAA's regional database. Specifically, we calculated rDHW following the general Equation (1):

(1) $$\text{rDHW}=\sum_{j=i-83}^{i}\left(\frac{{\mathrm{HS}}_{j}}{7}\right)$$

for *HS*_*j*_ (Coral Bleaching HotSpot value) ≥ 1, where *HS*_*j*_ is measured as the difference between in situ seawater temperature (daily mean or daily maximum) recorded between 6.5 and 8 m deep and the long‐term mean SST of the climatologically hottest month of the year, referred to as the Maximum Monthly Mean (MMM_SST_climatology). *HS*_*j*_ is calculated following the Equation (2a) for the rDHW_STmean, where *STmean* is the in situ daily mean seawater temperature recorded between 6.5 and 8 m deep, or the Equation (2b) for the rDHW_STmax, where *ST*_max_ is the in situ daily maximum seawater temperature recorded between 6.5 and 8 m deep:

(2a) $${\mathrm{HS}}_{j}={\mathrm{ST}}_{\text{mean}}-\mathrm{MMM}\_\mathrm{SST}\_\text{climatology}$$

(2b) $${\mathrm{HS}}_{j}={\mathrm{ST}}_{\max}-\mathrm{MMM}\_\mathrm{SST}\_\text{climatology}$$

DHW data for the Madinah‐Makkah region has been extracted from NOAA regional database: https://coralreefwatch.noaa.gov/product/vs/gauges/madinah_makkah.php. Seawater current data is visualized in polar diagrams for each location and season of each year by adapting the “windRose” function from the “openair” package in R (Carslaw and Ropkins 2012).

### 2.5 Survey of Microbial Therapy Validation Studies Conducted at the CPV

A structured survey of peer‐reviewed in situ microbial restoration studies conducted at the CPV was performed to evaluate the validation of the CPV as a functional research platform for coral microbiome‐based restoration.

## 3 Results and Discussion

### 3.1 Sustained Environmental Data Observations and Surveillance Platform

**FIGURE 3:**
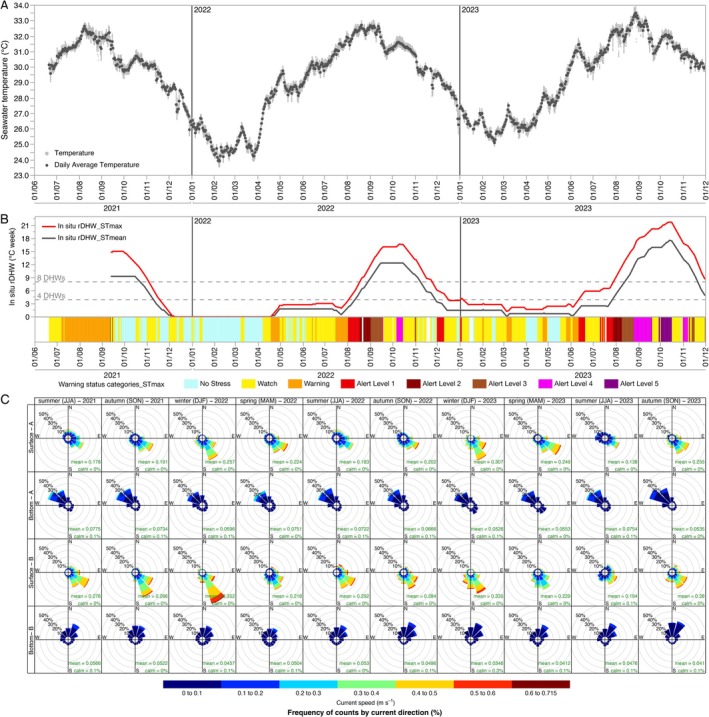
(A) In situ seawater temperature and daily mean temperature recorded between 6.5 and 8 m deep. (B) In situ rDHW, calculated using daily mean (rDHW_STmean) and daily maximum (rDHW_STmax) seawater temperature recorded between 6.5 and 8 m deep, and the warning status categories following NOAA criteria and calculated using the rDHW_STmax. (C) Seawater current direction and speed by season and year at the two data collection stations: Two upper rows correspond to surface and bottom layers from location A and the two lower rows to surface and bottom layers from location B (seawater current direction follows the oceanographic convention: Vectors show the direction to which currents are going).

In situ daily mean seawater temperature (measured at 0.5 m above the seafloor, between 6.5–8 m deep) ranged from 23.92°C and 25.11°C in winter 2022 and 2023, respectively, to 32.66°C, 32.73°C, and 33.50°C in summer 2021, 2022, and 2023, respectively. The minimum seawater temperature since the start of the monitoring was 23.54°C in winter 2022. The highest daily maximum temperature was 32.89°C in 2021, 33.05°C in 2022, and 33.92°C in 2023 (Figure 3A). Daily seawater temperature oscillation was lower than 1°C and overall median daily average salinity was 39.07 PSU. To assess the accumulated heat stress in our study site, we calculated the relative degree heat week (rDHW) based on the in situ daily mean (rDHW_STmean) and the daily maximum (rDHW_STmax) temperatures recorded at 6.5–8 m deep, as a measure of comparison to the NOAA DHW estimates, which rely on satellite sea surface temperature with 5 km^2^ resolution. The year 2023 was notably warmer than the previous 2 years, reflected in the highest rDHW_STmax, 21.8°C‐weeks, recorded on October 13, 2023 (Figure 3B), similar to the highest DHW reported by NOAA for the region (22.3°C‐weeks on October 18 for Al Madinah—Makkah region). Overall, in 2023, we recorded 19 consecutive weeks in which rDHW_STmax was higher than 8°C‐weeks with 31 days falling in the “Alert Level 4” and 18 days in the “Alert level 5” of the Coral Reef Watch's Bleaching Alert scale, following NOAA criteria; coinciding with a severe coral bleaching event at the study site and in the region that started in September 2023. Many coral colonies remained bleached at the end of October, as shown by the drone imagery (Figure 2C).

The main surface current direction was from NW to SE, following the predominant winds in the area, while bottom currents (1 m above the sea floor) were mostly from SE to NW in location A and from SW to NE in location B within our study site. Overall, surface currents seemed to be driven by predominant winds, while the currents at the bottom layers were driven by the seafloor topography. The strongest bottom currents were recorded during summer (June–August) months in both locations (with a maximum mean current speed of 0.078 m s^−1^ in summer 2021). Winter months were calmer (with a mean current speed ranging from 0.059 to 0.035 m s^−1^), with overall predominantly stronger currents in location A compared to B. Conversely, surface currents were stronger in winter months for both locations (with a maximum mean current speed of 0.35 m s^−1^ and with a peak current speed of 0.715 m s^−1^ in winter 2022) (Figure 3C).

The 3D photogrammetry model generated of the CPV (Figure 2B) provides a powerful biological baseline data to assess the structure and biodiversity of this reef area, document temporal changes, and evaluate the effect of future experimental interventions. Altogether, this well‐controlled platform provides a valuable surveillance opportunity to closely monitor coral bleaching events, spawning events, disease outbreaks in corals and other reef organisms, and the appearance of invasive species to allow timely actions to reduce the risk of spreading. It also provides a powerful tool for outreach and education about the research and restoration activities and technological development opportunities while raising population awareness on the current coral global crisis and biodiversity loss (Figure 1).

### 3.2 In Situ Validation of Microbial Restoration Strategies at the CPV

This section presents key previously published experimental outcomes generated at the CPV, including the published studies that validate the site's scientific capacity for in situ microbiome‐based coral restoration. The CPV offers a uniquely suited experimental environment for in situ validation and ecological risk assessment of microbial interventions under real‐world reef conditions. Its design includes close monitoring of both biotic and abiotic parameters, along with replicated experimental units representing varying levels of biodiversity, from individual coral colonies to small, ecologically integrated patches. This infrastructure enabled the first successful in situ probiotic application on corals, demonstrating the feasibility of experimentally restoring the native microbiome of corals through the external application of BMCs to the surroundings of *Pocillopora verrucosa* coral colonies (Delgadillo‐Ordoñez et al. 2024). Administering probiotics three times per week over a three‐month period led to measurable restructuring of the coral microbiome with no adverse effects on coral health or detectable off‐target effects in the surrounding seawater and sediment microbial communities. Notably, this in situ coral BMCs intervention also resulted in a shift in the gut microbiome of damselfish (*Dascyllus abudafur*) living in close association with treated corals, showing an increase in putative beneficial bacteria and a reduction in potential pathogens (Rosado et al. 2025).

In addition, the in situ application of BMCs on *Stylissa carteri* and *Callyspongia crassa* sponges resulted in a restructuring of their native microbiome and an enrichment of some BMC strains naturally found in *S. carteri* (Ribeiro et al. 2024). These pioneering field experiments suggest that coral BMCs application does not negatively affect the health of other key reef organisms and that sponges and fish can be enriched in certain beneficial microbes.

The outcomes of the in situ microbiome monitoring and restoration experiments provide field validation and risk assessment data to support the development of new microbial therapies to improve coral resilience and survival to be applied in restoration efforts (Figure 1). Ultimately, pilot tests help advance, validate, and boost the development technology that can overcome the challenging nature of these field experiments, for instance, by improving the coral health monitoring or automatizing the probiotics administration, as discussed below.

### 3.3 Future Outcomes: Ongoing Technology Deployment and Validation for In Situ Reef Restoration at the CPV

The link between technology and conservation is critical and presents an opportunity to conceive innovative solutions to environmental challenges while fostering their optimization and scaling. Unfortunately, the field of coral restoration shows a gap between the technological advancements indicated by patents, academic research, and the real needs of coral restoration practitioners (Roch et al. 2023). For instance, there is a pressing need for continuous, long‐term monitoring of coral reefs; however, the scale and temporal resolution pose some of the main challenges. The development of autonomous monitoring tools can help address these challenges. An underwater robot prototype capable of safely navigating and capturing high‐definition images is currently being tested to monitor the reef status in a semi‐autonomous manner and to enable near real‐time and on‐demand streaming of visual data (Figure S1).

AI‐assisted approaches are integrated for reef monitoring, allowing us to track coral diversity, health, and growth, and providing a baseline for comparison to an array of physico‐chemical measurements (flow fields, salinity, temperature, O_2_, pH, etc.). Standardized reef benthic surveys in defined time intervals will allow reef monitoring using ML‐assisted online platforms for reef survey data annotation from underwater imagery (Williams et al. 2019; Colin et al. 2024). Further, AI‐assisted approaches will be used to identify bacterial candidates associated with coral heat stress resilience by using coral microbiome sequencing and thermal tolerance data. These approaches mainly aim to assist the processing/filtering/subselection of data, such as the identification of bacteria that correlate with increased thermal tolerance using the machine learning framework (ML) Coracle (Staab et al. 2024). These bacteria are (i) coral probiotic candidates for testing and (ii) further provide biomarkers for integration into reef monitoring approaches. Portable technologies for effective microbial diversity assessments in marine environments were adapted onboard a research vessel in the CPV for coral sample processing (Jiménez et al. 2025). The workflow automated critical steps, including lysis, binding, washing, and elution, producing high‐quality DNA suitable for sequencing workflows, including full‐length 16S rRNA gene amplicon sequencing and long‐read shotgun metagenomics. In situ environmental sample processing for further characterization of microbial communities reduces limitations associated with sample transportation and storage in remote areas and allows for more accurate coral‐associated microbiome data, providing critical insights to monitor, protect, and restore marine ecosystems effectively. We are also currently testing underwater automated dispensers to improve the efficiency of coral probiotics delivery and overcome scaling‐up challenges. The designed autonomous dispensers (de Oliveira Filho et al. 2022) are capable of continuous operation for 30 days, with the possibility to adjust the dosage and agitation parameters.

National and international commitments to improve and retain healthy ecosystems (e.g., most recently the Kunming–Montreal declaration (Convention on Biological Diversity 2022)) have resulted in a major acceleration of ecosystem restoration globally, including for reefs (Suggett et al. 2024). Indeed, coral reef health and cover in the Red Sea are decreasing and remain at risk from intense anthropogenic pressures (Ziegler et al. 2019). Consequently, numerous mitigation, rehabilitation, and restoration activities are underway throughout the Red Sea—importantly, whilst ecological restoration can be operationally achieved from scales of local “home” reef sites (typically a few hectares) to entire reefs, the scale ultimately achieved governs the range of ecosystem services that can be retained or returned (Kaufman et al. 2021). Regardless, few restoration activities exceed the scale of a single hectare (Boström‐Einarsson et al. 2020; Lange et al. 2024). The CPV (0.5 ha) provides an ideal hub for evaluating the feasibility of restoration approaches, i.e., using restoration ecology experimentation (and research and development) to assess their success in a comparative framework and how they can advance ecological restoration activities both within Saudi Arabia and globally to increase in scale (Figure 1). For example, the world's largest reef restoration program (KAUST Coral Restoration Initiative, KCRI) operates over a 100 ha reef site located in the northern Red Sea, where corals are currently considered highly buffered against thermal stress (Osman et al. 2018) that is causing die‐off from mass coral bleaching events for reefs globally (e.g., Hughes et al. 2018). Resilience‐based reef restoration (Shaver et al. 2022) focuses on optimizing population‐level diversity; however, it is unlikely that the northern Red Sea will remain immune to the effects of thermal stress if climate change continues unaddressed. The surroundings of the CPV provided a testing ground for a suite of coral restoration technologies developed at KAUST (Schmidt‐Roach et al. 2023), which enable workflows that integrate assisted adaptation, such as selective breeding and assisted migration to increase stress tolerance. Resilience‐based restoration activities and in particular microbial interventions (i.e., the pioneering application of coral probiotics in situ) that either aid thermal tolerance or post‐stress recovery will be required for ecological restoration efforts to be more effective and sustained over space and time. The use of probiotics to boost coral growth and health in conventional coral nurseries, in situ and ex situ, should also be considered (Thatcher et al. 2022).

Cutting‐edge restoration strategies combined with state‐of‐the‐art technologies, such as robotic 3D printing and newly developed eco‐friendly biodegradable and biocompatible materials, are integrated into coral restoration efforts. With the aim to mimic the coral skeleton's composition, biocompatible calcium carbonate‐containing (CCP) resins are used as a 3D printing ink for printing and molding CCP coral copies of scanned coral species for larger‐scale coral restoration applications (Albalawi et al. 2021). The CPV functions as a living lab for testing and validating such advanced technologies for reef monitoring, microbiome research, and restoration interventions under field conditions.

## 4 Concluding Remarks

The establishment and validation of the CPV represents a timely and critical advancement in the field of coral reef conservation and restoration. As the field moves beyond controlled laboratory experiments, there is a pressing need for pilot‐scale in situ validation to enable the translation of promising tools (Peixoto et al. 2022) to actual implementation at medium (1 ha) and large (up to 100 ha) scales. Despite their potential, coral probiotics present unique challenges due to the high taxonomic and functional complexity of coral microbiomes, the intricacy of host‐microbe‐environment interactions, and the context dependency of microbial performance (Bourne et al. 2016; Leite et al. 2018; van Oppen and Blackall 2019).

In this context, continuous and comprehensive environmental and biological monitoring are crucial to inform experimental design, evaluate ecological responses, and generate high‐resolution datasets that support adaptive restoration strategies. The CPV provides a controlled yet ecologically realistic platform to meet this need. The data collected here will serve not only as a local baseline but also as a reference for global coral reef studies.

We envision the CPV as a long‐term, multi‐disciplinary research hub that enables the rigorous testing and refinement of microbial therapies and other cutting‐edge, coral reef‐assisted restoration‐guided technologies. As a reproducible model for integrated reef restoration, the CPV offers a pathway to accelerate the development, validation, and deployment of next‐generation interventions at meaningful ecological scales. Its continued use and similar pilot tests replicated worldwide will support the global shift toward science‐based, scalable solutions to confront the coral reef crisis.

## Author Contributions

**Neus Garcias‐Bonet:** conceptualization (equal), data curation (lead), formal analysis (lead), investigation (lead), methodology (lead), writing – original draft (lead), writing – review and editing (lead). **Helena Villela:** investigation (equal), methodology (equal), writing – review and editing (equal). **Francisca C. García:** data curation (equal), investigation (equal), methodology (equal), writing – review and editing (equal). **Gustavo A. S. Duarte:** investigation (equal), methodology (equal), writing – review and editing (equal). **Nathalia Delgadillo‐Ordoñez:** investigation (equal), methodology (equal), writing – review and editing (equal). **Inês Raimundo:** investigation (equal), methodology (equal), writing – review and editing (equal). **Yusuf C. El‐Khaled:** investigation (equal), methodology (equal), writing – review and editing (equal). **Erika P. Santoro:** investigation (equal), methodology (equal), writing – review and editing (equal). **Morgan Bennett‐Smith:** methodology (equal), writing – review and editing (equal). **Brian O. Nieuwenhuis:** investigation (equal), methodology (equal), writing – review and editing (equal). **João Curdia:** investigation (equal), methodology (equal), writing – review and editing (equal). **Brian Zgliczynski:** investigation (equal), methodology (equal), writing – review and editing (equal). **Clinton Edwards:** investigation (equal), methodology (equal), writing – review and editing (equal). **Stuart Sandin:** investigation (equal), methodology (equal), writing – review and editing (equal). **Eslam O. Osman:** investigation (equal), methodology (equal), writing – review and editing (equal). **Ronell Sicat:** investigation (equal), methodology (equal), writing – review and editing (equal). **Alexander Przybysz:** methodology (equal), writing – review and editing (equal). **Alexandre S. Rosado:** conceptualization (equal), writing – review and editing (equal). **Burton H. Jones:** investigation (equal), writing – review and editing (equal). **Francesca Benzoni:** conceptualization (equal), writing – review and editing (equal). **Michael L. Berumen:** conceptualization (equal), writing – review and editing (equal). **Khaled Salama:** invesigation (equal), writing – review and editing (equal). **Shinkyu Park:** investigation (equal), writing – review and editing (equal). **Manuel Aranda:** investigation (equal), writing – review and editing (equal). **Carlos M. Duarte:** investigation (equal), writing – review and editing (equal). **Sebastian Schmidt‐Roach:** investigation (equal), methodology (equal), writing – review and editing (equal). **Charlotte A. E. Hauser:** investigation (equal), methodology (equal), writing – review and editing (equal). **Tadd Truscott:** investigation (equal), writing – review and editing (equal). **David J. Suggett:** investigation (equal), writing – review and editing (equal). **Christian R. Voolstra:** conceptualization (equal), investigation (equal), writing – review and editing (equal). **Susana Carvalho:** conceptualization (equal), investigation (equal), writing – review and editing (equal). **Raquel S. Peixoto:** conceptualization (lead), funding acquisition (lead), investigation (equal), methodology (equal), writing – original draft (equal), writing – review and editing (equal).

## Conflicts of Interest

The authors declare no conflicts of interest.

## Supporting information


Figure S1

## Data Availability

Environmental data, orthomosaic files, and CPV model are available in the Zenodo repository: https://doi.org/10.5281/zenodo.15575058.
