# Supplementary material for: The Coral Probiotics Village: An Underwater Laboratory to Tackle the Coral Reefs Crisis
Source: Ecol Evol. 2025 Jul 4;15(7):e71558. doi: 10.1002/ece3.71558 (PMC12231234; doi:10.1002/ece3.71558)
Supplement: Supplementary file 1 — Figure S1 [file ECE3-15-e71558-s001.docx]

**SUPPORTING INFORMATION**

**The Coral Probiotics Village:**

**An underwater laboratory to tackle the coral reefs crisis**

Neus Garcias-Bonet^1*^, Helena Villela^1^, Francisca C. García^1^, Gustavo A. S. Duarte^1^, Nathalia Delgadillo-Ordoñez^1^, Inês Raimundo^1^, Yusuf C. El-Khaled^1^, Erika P. Santoro^1^, Morgan Bennett-Smith^1,2^, Brian O. Nieuwenhuis^1^, João Curdia^1^, Brian Zgliczynski^3^, Clinton Edwards^3^, Stuart Sandin^3^, Eslam O. Osman^1^, Ronell Sicat^4^, Alexander Przybysz^5^, Alexandre S. Rosado, Burton H Jones^1^, Francesca Benzoni^1^, Michael L Berumen^1^, Khaled Salama^5^, Shinkyu Park^5^, Manuel Aranda^1^, Carlos M. Duarte^1^, Sebastian Schmidt-Roach^1^, Charlotte A. E. Hauser^6,7^, Tadd Truscott^8^, David J. Suggett^9,10^, Christian R. Voolstra ^11^, Susana Carvalho^1^, Raquel S. Peixoto^1*^

### **Supplementary figure**

**Figure S1**. A. Image of the underwater robot prototype. B. Visualization of the 3D reconstruction derived from in situ imaging data collected in the CPV.
